# Incidence du carcinome hépatocellulaire lors de l'infection chronique par le virus de l'hépatite B

**DOI:** 10.11604/pamj.2015.20.167.6227

**Published:** 2015-02-23

**Authors:** Rénovat Ntagirabiri, Bélyse Munezero, Hippolyte Kaze, Eugène Ndirahisha, Sébastien Manirakiza

**Affiliations:** 1 Centre Hospitalo-Universitaire de Kamenge, Département de Médecine Interne, Bujumbura, Burundi; 2 Centre des maladies du tube digestif et du foie, Bujumbura, Burundi; 3 Centre Hospitalo-Universitaire de Kamenge, Département de Radiologie, Bujumbura

**Keywords:** Carcinome hépatocellulaire, Cirrhose, AgHBs, virus de l'hépatite B, fibrose hépatique, hepatocellular carcinoma, cirrhosis, AgHBs, hepatitis B virus, liver fibrosis

## Abstract

**Introduction:**

Le virus de l'hépatite B (VHB) est incriminé comme cause de cancer primitif du foie. Le stade de fibrose et d'autres facteurs environnementaux et génétiques seraient intriqués. Le but de notre travail était de déterminer l'incidence du carcinome hépatocellulaire (CHC) lors d'une infection chronique par le VHB et estimer le risque relatif (RR) de CHC lié au stade de la fibrose hépatique.

**Méthodes:**

Étude prospective de suivi d'une cohorte de patients porteurs chroniques du VHB sur une période de 5 ans (2009 à 2014). Etaient inclus les patients consécutifs qui ont subi un dosage de la charge virale B, une évaluation de la fibrose hépatique et un suivi régulier de tous les 6 à 12 mois par une échographie hépatique.

**Résultats:**

Au total 194 patients ont été retenus. L’âge moyen était de 39,1 ans. Parmi eux 112 étaient des hommes. L'incidence cumulée de CHC a été de 8,8% dans la population d’étude soit une incidence annuelle de 1,8%. Selon le stade de fibrose, 31 patients avaient une fibrose sévère ou une cirrhose (score Fibrotest >0,73). Parmi eux, l'incidence cumulée de CHC était de 35,5% soit une incidence annuelle estimée à 7,10%. Parmi 163 patients ayant une fibrose mineure, l'incidence cumulée de CHC était de 3,7% soit une incidence annuelle de 0,7%. Le RR lié à la cirrhose était de 9,7; IC 95%: (3,8-24,1%).

**Conclusion:**

Le VHB expose au CHC jusqu’à 10 fois. La fibrose sévère et la cirrhose constituent des facteurs prédictifs de CHC chez le porteur chronique du VHB. Evaluer systématiquement la fibrose pour traiter précocement les malades pourra prévenir l’évolution vers la cirrhose et par là réduire la survenue du CHC.

## Introduction

Le carcinome hépatocellulaire (CHC) est une complication redoutable des maladies chroniques du foie. Il constitue 70-90% des cancers primitifs du foie [1, 2]. Il est la troisième cause de décès liés au cancer et son fardeau va en augmentant [2–4]. L’épidémiologie du CHC dans le monde est très contrastée [5, 6]. Il existe des zones géographiques de forte endémie, principalement l'Afrique et l'Asie. On estime que 80% des cas de CHC dans le monde y surviennent. Dans les régions de forte endémie, le CHC est principalement associé aux maladies chroniques du foie d'origine virale, en particulier le virus de l'hépatite B (VHB) dont la contamination survient à la naissance ou lors des premières années de la vie. Il est potentialisé par l'aflatoxine B1 qui est aussi un carcinogène puissant et par le virus de l'hépatite C (VHC) [7]. En plus de ces facteurs de risque avérés, des spécificités environnementales et ethniques joueraient également un rôle non moindre dans l'incidence du CHC. Toutefois, en Afrique subsaharienne, très peu de travaux relatifs à l'intrication de ces facteurs de risque ont été réalisés. Dans notre pays, aucun travail n'a étudié l'association et la force de liaison qui existeraient entre le portage chronique du VHB et la survenue du CHC. Il nous a paru alors important de mener une étude dont le but était de déterminer l′incidence du CHC lors d'une infection chronique par le VHB et d'estimer le risque relatif (RR) de CHC lié au stade de la fibrose hépatique.

## Méthodes

**Type d’étude:** il s'agit d'une étude prospective de suivi de cohorte ayant inclus des patients porteurs chroniques du VHB durant la période allant de Juin 2009 à Mai 2014.

**Critères d'inclusion:** étaient éligibles à l’étude tous les patients porteurs chroniques de l'AgHBs. Le portage chronique était défini par un AgHBs positif depuis plus de 6 mois. Ont été retenus pour analyse les patients qui ont régulièrement subi une échographie du foie tous les 6 à 12 mois durant la période de l’étude et qui ont eu une évaluation de la fibrose hépatique et le dosage de l'ADN virale B. Les dosages virologiques et les paramètres du Fibrotest ont été réalisés dans le Laboratoire Cerba à Paris, France.

**Evaluation de la fibrose:** tous les malades ont eu une évaluation de la fibrose basée sur trois scores sanguins à savoir le Fibrotest, l'Aspartate transaminase to platelet ratio index (APRI) et le score FIB-4. Certains malades ont eu en plus le Fibroscan. Les appareils utilisés étaient les Fibroscan 402 ou 512 Echonsens, Paris, France. Un résultat Fibroscan considéré comme valide était caractérisé par un minimum de 10 mesures valides, un taux de réussite de 66% et un rapport de l'interquartile range (IQR) sur la médiane <33%. La médiane des mesures était considérée comme l’élasticité du foie. Les patients ont été repartis en deux bras: ceux ayant la cirrhose et ceux n'ayant pas la cirrhose. L'existence d'une cirrhose était retenue devant un score Fibrotest supérieur ou égal à 0,73; un score APRI >1,5 et un score FIB-4 >3,5.

**Moyens de dépistage du CHC:** Moyens de dépistage du CHC: les malades ayant un score Fibrotest supérieur ou égal à 0,73 avaient à faire au moins une échographie tous les 6 mois et ceux ayant un score Fibrotest inférieur à 0,73 au moins une échographie tous les 12 mois. Les résultats des échographies acceptées pour l’étude étaient celles réalisées par des praticiens ayant des compétences et un minimum de cinq ans d′expérience en échographie. Deux centres ont été retenus: le CHU Kamenge et le CEMADIF. Les appareils utilisés étaient tous de haute résolution. C’étaient principalement les appareils HITASHI EUB 625 équipés d′une sonde linéaire et d′une sonde sectorielle à balayage électronique couplé à un module doppler couleur et doppler pulsé.

**Critères diagnostiques du CHC:** le diagnostic de CHC était retenu sur des critères échographiques et évolutifs. Le principal critère diagnostique était l'apparition à l’échographie d'une ou de plusieurs lésions focales hépatiques. La lésion devait augmenter progressivement de volume au cours des 3 à 24 mois suivant le diagnostic. Secondairement, une élévation progressive de la valeur de l'AFP était considérée comme un élément renforçant le diagnostic.

**Analyse des résultats:** les logiciels « Le Sphinx Plus2 et SPSS for Windows 10.0 » ont servi pour la saisie et l′analyse des données. Certaines valeurs des résultats ont été exprimées assorties de leurs intervalles de confiance à 95% (IC). Le test du Chi carré a été utilisé pour comparer les proportions. La valeur de p < 0,05 a été considérée comme statistiquement significative. L'IC du risque relatif (RR) a servi de test statistique.

## Résultats

**Caractéristiques de la population étudiée:** 194 patients porteurs du VHB ont été retenus pour analyse. 82 malades (42,3% IC:(35-49%)) étaient de sexe féminin et 112 (57,7% IC:(51-65%)) de sexe masculin. L′âge moyen de la population totale était de 39,1 ans ±10,5 ans. Selon la réplication virale, 132 patients (68%) avaient une virémie inférieure à 2000 UI/ml et 179 patients (92,2%) avaient un AgHBe négatif.

**Incidence du CHC dans la population étudiée:** au cours de la période de suivi, le nombre de cas de CHC était de 17 sur les 194 malades. L'incidence cumulée sur 5ans était de 8,8% IC:(4-13%). L'incidence annuelle était estimée à 1,8%.

**Incidence du CHC chez les patients sans cirrhose:** le groupe de patients ayant un score Fibrotest <0,73 était composé de 163 malades soit 83,7% de la population d’étude. Parmi eux, 76 (46,6%) étaient de sexe féminin et 87 (53,4%) de sexe masculin. La moyenne d’âge était de 39,2 ans. Sur les 5 ans de suivi, 6 patients parmi les 163 malades ont développé un CHC soit une incidence cumulée de 3,7% IC:(1-7%). L'incidence annuelle était estimée à 0,7% dans ce groupe.

**Incidence du CHC chez les patients avec cirrhose:** le groupe de patients ayant un score Fibrotest ≥0,73 était composé de 31 patients soit 16,3% de la population d’étude. Parmi eux, 25 patients (80,6%) étaient de sexe masculin et 6 patients (19,4%) de sexe féminin. La moyenne d’âge était calculée à 38,6 ans. Durant la période de suivi, 11 patients ont développé un CHC soit une incidence cumulée de 35,5% IC:(21-53%). L'incidence annuelle était estimée à 7,1%.

**Risque relatif de CHC lié à la présence de la cirrhose:** la différence entre les taux d'incidence du CHC dans les deux groupes était statistiquement significative: p < 0,001. Le RR de CHC lié à la présence de cirrhose était de 9,7. Le Tableau 1 donne les détails sur les incidences du CHC dans les deux groupes, le RR et son IC 95%.

**Tableau 1:** Incidence du CHC et RR lié à la cirrhose lors du portage chronique de l'AgHBs

Facteur de risque étudié: la cirrhose	Population d’étude	Cas de CHC	Incidence cumulée sur 5 ans	Incidence annuelle	RR	IC 95% du RR
Présente	31	11	35,5%	7,10%	9,7	(3,8-24,1)
Absente	163	6	3,7%	0,74%		
Total	194	17	8,8%	1,8%	-	-

CHC: Carcinome hépatocellulaire; RR: Risque relatif; IC95%: Intervalle de confiance à 95%

## Discussion

L’étude a permis d'estimer l'incidence annuelle de CHC en cas de portage chronique du VHB à 1,8%. Cette incidence passe de 0,74% en l'absence de cirrhose à 7,1% en cas de cirrhose. Le risque relatif lié à la cirrhose était alors de 9,7. Il s'agit des résultats qui corroborent l'existence d'une association entre le portage chronique du VHB et le CHC dans notre pays. Cette liaison a été décrite pour la première fois par Blumberg et al en 1975 [8]. Dans notre étude, le diagnostic de CHC était principalement basé sur des critères échographiques et évolutifs. L'objectif en utilisant l’échographie était de ne pas s’éloigner de la vraie vie quotidienne de pratique médicale au Burundi. D'abord, l’échographie est un procédé de dépistage simple, moins couteux, efficace et disponible. C'est pourquoi beaucoup de travaux de dépistage de CHC ont été réalisés grâce à l’échographie [7, 9, 10]. Ensuite, dans notre pays, la biopsie n'est pas de pratique courante. C'est un acte invasif mal accepté par les patients. Le nombre de pathologistes est aussi limité. Ils étaient au nombre de deux pour tout le pays durant la période de l’étude. Cela était aussi un facteur limitant la biopsie. Quant au diagnostic de la cirrhose, il était basé sur une combinaison de méthodes non invasives d’évaluation de la fibrose hépatique largement utilisées dans le monde. Préalablement validées pour l'hépatite virale C, ces méthodes non invasives sont aujourd'hui de plus en plus utilisées en cas d'hépatite virale B, surtout dans les pays endémiques. Une étude récente burkinabaise a montré leur validité en cas de VHB, comparées à la biopsie [11]. De même pour la fibrose, bien que le gold standard reste la biopsie hépatique, il s'agirait d'un Gold standard imparfait car elle a des limites du fait des erreurs d’échantillonnage, de la taille de la biopsie (5 à 30mm) et de la variabilité intra et inter-observateur. Ainsi, la biopsie hépatique présenterait une erreur moyenne de 30%, d'où l'on parle alors de “Gold Standard imparfait” [12].

Nos résultats montrent aussi qu'en plus du risque de CHC lié au VHB lui même, la présence d'une fibrose sévère et notamment de la cirrhose est un facteur de risque majeur qui multiplie par 9 environ la survenue de CHC. En effet, il est admis que le VHB serait responsable de 55% de CHC au niveau mondial et de 89% de CHC dans les pays endémique [13–15]. Ce risque est très élevé chez l'homme que chez la femme, ce qui est même trouvé par les auteurs africains [16, 17]. Parkin et al rapportent un RR variant de 5 à 98 selon les études [18]. Même s'il est admis que le VHB entraine un CHC avec un risque élevé, il est important de noter que l'incidence de CHC dans le groupe sans fibrose était de 0,7% dans notre étude. De nos résultats, on peut extrapoler et dire qu'en traitant systématiquement les malades porteurs du VHB actif, on évitera la progression vers la fibrose sévère et la cirrhose et on réduira ainsi le risque de CHC d'environ 10 fois. En somme, notre étude apporte une contribution non moindre dans les connaissances des facteurs prédictifs de CHC en cas d'infection par le VHB dans la région subsaharienne spécifiquement dans notre pays où les travaux y relatifs sont rares. Il est alors recommandable que l’évaluation de la fibrose soit intégrée dans la pratique courante des gastroentérologues des régions endémiques spécifiquement ceux de la région subsaharienne. Cela permettra de sélectionner les malades porteurs chroniques du VHB actif pour les traiter à temps en vue d’éviter la progression vers la fibrose sévère et la cirrhose, facteur de risque avéré de CHC. Cette recommandation parait indispensable d'autant que la prise en charge du CHC est trop difficile en Afrique subsaharienne en général et au Burundi en particulier. En effet, une prise en charge du CHC dans un but curatif est quasi inexistante [19].

## Conclusion

Le VHB est un oncogène puissant. En sa présence, la cirrhose multiplie par 9,7 le risque de CHC. Il est important de promouvoir l’évaluation de la fibrose hépatique chez les porteurs chroniques de l'AgHBs pour sélectionner les porteurs actifs et les traiter précocement. La prévention de l’évolution vers la cirrhose réduira ainsi le risque de CHC par 10 environ.
